# Lipid droplets: a candidate new research field for epithelial ovarian cancer

**DOI:** 10.3389/fphar.2024.1437161

**Published:** 2024-07-01

**Authors:** Shiro Koizume, Tomoko Takahashi, Yohei Miyagi

**Affiliations:** ^1^ Molecular Pathology and Genetics Division, Kanagawa Cancer Center Research Institute, Yokohama, Japan; ^2^ Department of Pathology, Kanagawa Cancer Center Hospital, Yokohama, Japan

**Keywords:** ovarian clear cell carcinoma, clear cell renal cell carcinoma, lipid droplet, fatty acid oxidation, lipophagy, poly ADP-ribose polymerase inhibition

## Abstract

Ovarian clear cell carcinoma (OCCC) is a histological subtype that constitutes approximately 20% of epithelial ovarian cancer cases in Asian countries, but has a relatively low incidence in Western countries. Meanwhile, clear cell renal cell carcinoma (ccRCC) is a major subtype of kidney cancer. OCCC and ccRCC resemble one another histologically and have clear cytoplasmic appearances. Studies have revealed some genetic similarities between OCCC and ccRCC. However, information regarding common biological background factors between these cancers remains scarce. For example, accumulation of cellular lipid droplets was shown to play a crucial role in ccRCC progression, while similar information is lacking for OCCC. In this perspective article, we propose that lipid droplets may be candidates for future exploration to better understand the common biological backgrounds between OCCC and ccRCC, potentially leading to subtype-specific treatment strategies. We further discuss the relationship between poly ADP-ribose polymerase inhibition treatment and lipid metabolism because this therapeutic strategy has attracted considerable attention as a treatment for epithelial ovarian cancer.

## 1 Introduction

Epithelial ovarian cancer (EOC) is the most lethal gynecological disease worldwide. Globally, 313,959 new EOC cases were diagnosed and 207,252 deaths were recorded in 2020 (Huang et al., 2022). EOC can be classified into four histological subtypes: serous, endometrioid, mucinous, and clear cell (Torre et al., 2018). Ovarian clear cell carcinoma (OCCC) has an incidence of approximately 20% in Asian countries and limited European countries, but is rare in most Western countries (Sung et al., 2014; Kato, 2020). OCCC cells are glycogen-rich with a clear cytoplasm. Because OCCC is aggressive and exhibits drug resistance (Kato, 2020), it is regarded as an intractable cancer.

The appearance of OCCC resembles that of clear cell renal cell carcinoma (ccRCC) (Ji et al., 2018; Ackroyd et al., 2023), a histological subtype found in approximately 80% of kidney cancer cases. Studies have revealed molecular and genetic similarities and differences between OCCC and ccRCC (Ji et al., 2018; Ackroyd et al., 2023). Further exploration of these factors may lead to not only greater understanding of the similar histological appearances between OCCC and ccRCC but also the generation of common management strategies for cancer types with clear cell histological appearances.

Many studies have revealed the crucial roles of dysregulated lipid metabolism in multiple cancer types (Bian et al., 2020), and new insights continue to be gained, including the involvement of fatty acid synthesis in breast cancer metastasis (Ferraro et al., 2021), ferroptosis resistance in glioblastoma (Minami et al., 2023), ferroptosis resistance in hepatocellular carcinoma (Li et al., 2024), and citrate transport-driven activation of lipogenesis and fatty acid oxidation (FAO) in pancreatic cancer cells (Zhang et al., 2023). Extensive cellular lipid uptake and synthesis, followed by lipid droplet (LD) formation, can contribute to cancer progression (Koizume and Miyagi, 2016; Cruz et al., 2020). Accumulated LDs play crucial roles in the expression of ccRCC phenotypes, such as cell motility (Chen et al., 2022; Quan et al., 2023), invasiveness (Chen et al., 2022), epithelial-to-mesenchymal transition (Chen et al., 2022), and resistance to cell death (Miess et al., 2018; Zhou et al., 2023). The LDs in ccRCC cells can also be catabolized by neutral lipases to release oleic acid on exposure to serum starvation and hypoxia (SSH), thereby maintaining cellular lipid homeostasis (Ackerman et al., 2018). Thus, both stored and released fatty acids (FAs) can contribute to ccRCC progression.

In contrast to ccRCC, little is known about lipid metabolism in OCCC cells. Indeed, there is scarce information on LD levels in OCCC (Cruz et al., 2020), and it remains unclear whether OCCC has higher LD levels than other histological subtypes of EOC. We recently reported that SSH triggers lipophagy for degradation of LDs in OCCC cells (Koizume et al., 2022). This LD catabolism synergistically activates multiple genes, including *ICAM1* and *CD69*, through activation of transcription factor NFκB binding to their promoter regions (Koizume et al., 2022). The proteins encoded by these genes lead to malignant phenotypes, such as apoptosis resistance (Koizume et al., 2015) and epithelial-to-mesenchymal transition with assistance of extracellular fibronectin (Koizume et al., 2023). Thus, we hypothesized that LDs may play major roles in OCCC progression, similar to the case for ccRCC.

## 2 Effect of FA oxidation on cancer progression and its correlation with ccRCC and OCCC

Cancer cells can utilize FAs received from their environments, including the bloodstream (Koizume and Miyagi, 2016), cancer-associated fibroblasts (Hwang et al., 2022), reactive astrocytes (Parida et al., 2023), and adipocytes (Nieman et al., 2011), and/or synthesized by themselves (Koizume and Miyagi, 2016; Cruz et al., 2020). FAs are a source of ATP produced through FAO in mitochondria, followed by oxidative phosphorylation. Carnitine palmitoyl transferase 1A (CPT1A) is the rate-limiting enzyme for FA transportation from the cytoplasm into mitochondria via carnitine (Liang, 2023). CPT1A is considered a therapeutic target in cancer (Zeng et al., 2016; Liang, 2023). Indeed, FAO inhibition was shown to block the growth of glioma (Pike et al., 2011), non-clear cell EOC (Nieman et al., 2011; Sawyer et al., 2020), gastric cancer (Wang et al., 2020), and myeloma (Tirado-Vélez et al., 2012) cells. A recent study further revealed that FAO contributes to metastasis of ccRCC through histone acetylation (Shi et al., 2024).

In contrast, FAO blockade can promote cancer cell growth. Studies have provided evidence that FAO can suppress aggressiveness in multiple cancer types (Zhang et al., 2017), hepatocellular carcinoma (Ma et al., 2021), and pancreatic cancer (Kim et al., 2023). This suppressive effect of FAO also functions in some cancer cells exposed to hypoxia, because the supply of molecular oxygen required for oxidative phosphorylation is restricted (Zhang et al., 2017; Kim et al., 2023). Thus, the way in which FAO functions for expression of malignant phenotypes may depend on the cell type and/or context.

## 3 Effect of LD on cancer progression and its correlation with ccRCC and OCCC

Excess FAs are converted to their esterified form and then incorporated into LDs for storage. FAs are released from LDs by lipophagy (Roy et al., 2017) and neutral lipolysis (Ackerman et al., 2018) when required and subsequently catabolized via FAO in cancer cells. However, LDs are not simply lipid storage compartments. Instead, LDs can contribute to cancer cell progression through multiple mechanisms, including elimination of reactive oxygen species and maintenance of endoplasmic reticulum homeostasis (Qiu et al., 2015; Koizume and Miyagi, 2016; Cruz et al., 2020). Indeed, high levels of cellular LDs (Cruz et al., 2020) and the importance of LDs over FAO has been demonstrated for ccRCC cells (Du et al., 2017; Xu et al., 2020; Zhou et al., 2023). Multiple studies have shown that FAO is suppressed in ccRCC cells to enhance LD generation with augmentation of the Warburg effect (Du et al., 2017; Courtney et al., 2018; Miess et al., 2018). In general, LD anabolism predominantly contributes to ccRCC progression, rather than FAO. However, it remains unclear whether this holds true for OCCC progression because of a lack of published data.

## 4 Effect of FAO inhibition on OCCC cell growth *in vitro*

**FIGURE 1:**
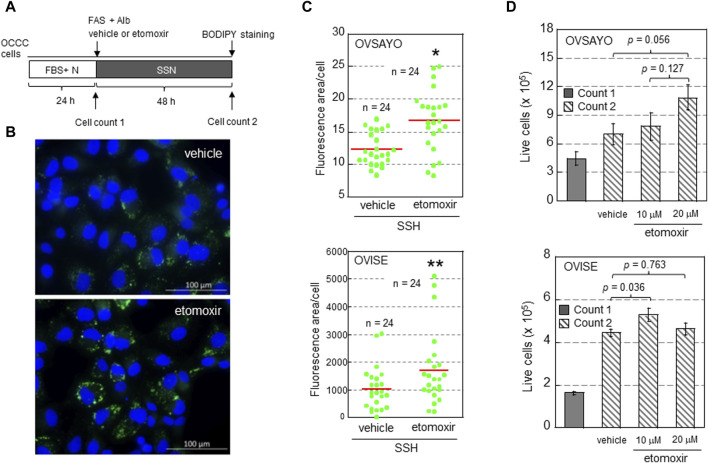
**(A)** Scheme of the assay. Following culture under normoxia (N) in medium supplemented with serum (FBS+), the effect of etomoxir (0, 10, or 20 μg/mL) and fatty acid supplement (FAS) (0.3%)-albumin (Alb) on the viability of OCCC cells cultured under serum starvation and normoxia (SSN) for 48 h was examined. **(B)** Typical Images of LDs (green) in OVISE cells cultured under SSN in medium supplemented with FAS-Alb in the presence of vehicle or etomoxir. The nuclei (blue) were counterstained with DAPI. **(C)** Effect of (+)-etomoxir (Cayman Chemicals) on LD levels in OCCC cells cultured under SSN in medium supplemented with FAS-Alb for 48 h. The LD levels were quantified by ImageJ software. The stained area was evaluated in the indicated number of images (green dots) acquired from three (OVSAYO) and two (OVISE) independent replicates and normalized to the number of cells (nuclei) in each image. Red bars: mean. *p < 0.001 versus vehicle, **p = 0.041 versus vehicle, by a *t*-test (OVSAYO) or the Mann–Whitney *U*-test (OVISE). **(D)** Effect of etomoxir treatment on the viability of OCCC cells cultured under SSN in medium supplemented with FAS-Alb for 48 h. Data are shown as mean ± SD (*N* = 3). *p* values were calculated by one-way ANOVA using SPSS Statistics 19.

To determine whether FAO facilitates or suppresses the growth of OCCC cells, we examined the effect of FAO inhibition on cell viability and LD levels in the presence of exogenous FAs (Figure 1). OCCC cells were cultured in serum-free medium supplemented with albumin and water-soluble fatty acid supplement (FAS) as previously described (Koizume et al., 2015; Koizume et al., 2022) (Figure 1A). The effect of etomoxir, a CPT1A inhibitor, on cell growth under serum starvation and normoxia (SSN) was elucidated by cell counting (trypan blue exclusion assay) and LD staining with fluorescent BODIPY dye as described (Koizume et al., 2022) (Figures 1A, B). We found that CPT1A inhibition increased the cellular LD level (Figures 1B, C), presumably because fatty acid retention in the cytoplasm shifted the equilibrium between LD generation and FAO to the former. This was associated with an increasing trend in OCCC cell growth (Figure 1D). These findings suggest that LDs, rather than FAO, are associated with OCCC cell viability, possibly due to LD-driven elimination of toxic effects, such as the generation of reactive oxygen species and serum deprivation-induced stresses. This is in contrast to the pro-survival effect of FAO associated with NADPH production in gliomas (Pike et al., 2011) and gastric cancer (Wang et al., 2020). The molecular mechanisms defining the relative importance of LD generation and FAO between these cancer cells are currently unclear.

## 5 Effect of MAP1S depletion on LD levels in OCCC cells

**FIGURE 2:**
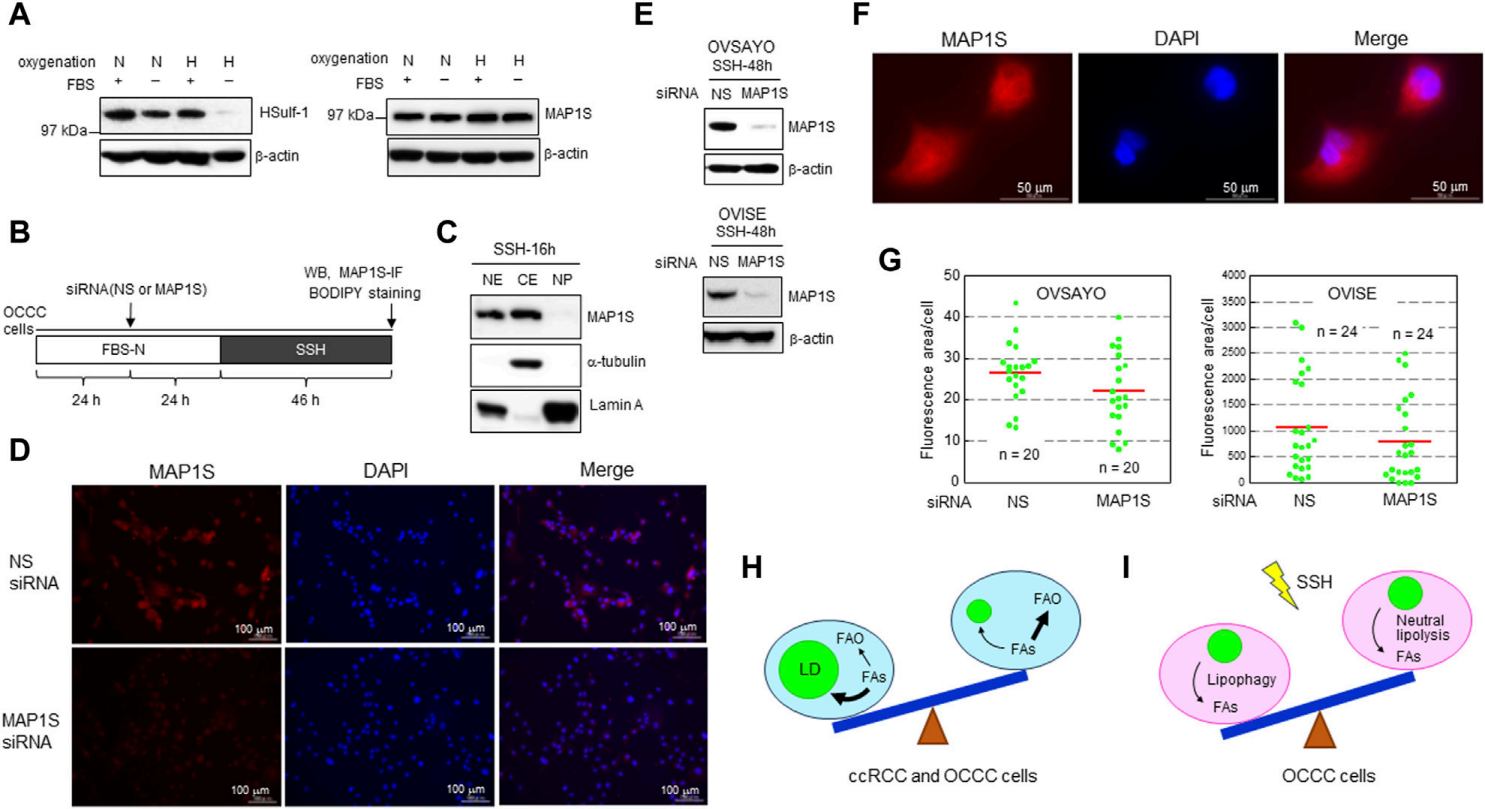
**(A)** Western blot analysis of the indicated proteins in OVSAYO cells exposed to the indicated culture conditions for 48 h. The primary antibodies were an anti-MAP1S rabbit polyclonal antibody (Proteintech) and an anti-HSulf-1 rabbit polyclonal antibody (CUSABIO). FBS: 10% fetal bovine serum; N, normoxia (ambient air); H, hypoxia (1% O_2_). β-Actin was examined as a protein loading control. **(B)** Scheme of the assay. Following culture under normoxia (N) in medium supplemented with serum (FBS+), the effect of MAP1S-knockdown on LD levels in OCCC cells cultured under SSH was examined by western blotting (WB), immunofluorescence (IF), and BODIPY staining. A MAP1S siRNA was purchased from Santa Cruz Biotechnology. **(C)** Western blot analysis of MAP1S expression in the cytoplasmic and nuclear fractions of OVSAYO cells cultured under the indicated conditions was performed as previously described (Koizume et al., 2022). NE, nuclear extract; CE, cytoplasmic extract; NP, nuclear pellet. Cytoplasmic (α-tubulin) and nuclear (lamin A) markers are shown. **(D)** Immunofluorescence staining of MAP1S in OVSAYO cells cultured as described for **(B)**. The primary anti-MAP1S antibody was the same as that used in the western blotting. **(E)** Western blotting analysis of MAP1S-knockdown in OCCC cells cultured under the indicated conditions. **(F)** Magnified version of the MAP1S immunofluorescence staining shown in **(D)**. **(G)** Effect of MAP1S knockdown on LD levels in OCCC cells cultured under SSH. The LD levels were quantified by ImageJ software. The stained area was evaluated for the indicated number of images (green dots) acquired from three (OVSAYO) and two (OVISE) independent replicates. Red bars: mean. *p* = 0.11 versus vehicle for OVSAYO, *p* = 0.25 versus vehicle for OVISE, by the Mann–Whitney *U*-test. **(H)** Seesaw model for FA metabolism in clear cell carcinoma cells. Bold arrows indicate predominant pathways. **(I)** Seesaw model for LD catabolism in OCCC cells exposed to SSH.

We previously showed that LD catabolism in OCCC cells in response to SSH exposure is mediated by lipophagy (Koizume et al., 2022). This mechanism is similar to the LD catabolism observed in ccRCC cells exposed to SSH to mitigate the toxic effect of excess FAs (Ackerman et al., 2018). Lipophagy is mediated by human sulfatase-1 (HSulf-1) in EOC cells (Roy et al., 2017). Meanwhile, microtubule-associated protein 1S (MAP1S) contributes to lipophagy in ccRCC cells (Xu et al., 2016). MAP1S binds to microtubules to facilitate autophagosomal biogenesis (Xie et al., 2011; Yue et al., 2017). Thus, we examined the effect of these potential lipophagy regulators on SSH-driven LD catabolism in OCCC cells. First, we examined the expression levels of these proteins by western blotting. HSulf-1 showed considerable expression in OCCC cell line OVSAYO cells under various oxygenation and serum supplementation conditions, but its expression was dramatically decreased under SSH (Figure 2A). In contrast, OVSAYO cells expressed MAP1S protein under all cell culture conditions (Figure 2A). Next, we examined the effect of MAP1S on LD degradation under SSH. If MAP1S participates in SSH-driven LD catabolism, the LD level should increase in response to MAP1S knockdown. Thus, we used an RNA interference approach to determine the effect of MAP1S expression on the LD level (Figure 2B). Western blotting (Figure 2C) and immunofluorescence (Figures 2D, F) analyses revealed that MAP1S existed in both the nucleus and the cytoplasm in OVSAYO cells, consistent with the subcellar localization data in a public database (The Human Protein Atlas, https://www.proteinatlas.org). Knockdown of MAP1S expression (Figures 2D, E) did not affect the LD levels in either OVSAYO cells or OVISE cells, another OCCC cell line, under SSH (Figure 2G).

## 6 Relationship between lipid metabolism and poly ADP-ribose polymerase inhibition in cancer cells

Inhibition of poly ADP-ribose polymerase (PARP) by small molecule inhibitors such as Olaparib is an important therapeutic strategy for EOC based on the synthetic lethality concept for impaired DNA repair machinery in the presence of *BRCA* gene mutations (Maiorano et al., 2023). In this section, we describe the published literature on the relationship between PARP inhibition (PARPi) therapy for cancer and lipid metabolism.

Despite a lack of published data on the relationship between PARPi therapy and lipid metabolism in *BRCA*-mutation-positive cancer types, a few studies have indicated that PARPi is effective in a *BRCA* mutation-independent manner, as described below. Indeed, *BRCA* mutations are rare in glioblastoma cells. However, PARPi functions in this cancer type through BRCAness, a phenotype expressed in sporadic cancers with similar biochemical pathways to familial cancers with *BRCA* mutations (Turner et al., 2004). Glioblastoma cells can evade PARPi-driven tumor suppression by metabolic reprogramming through LD generation followed by FAO (Majuelos-Melguizo et al., 2022). Meanwhile, PARPi can enhance oleic acid treatment-driven LD accumulation in mouse hepatoma cell line Hepa1-6 cells through lipogenic gene activation (Pang et al., 2018). These findings imply that clinical application of PARPi necessitates management of metabolic disease. In contrast, PARPi decreases cholesterol biosynthesis in ccRCC cells to block malignancy (Karpova et al., 2021). Currently, the relationship between lipid metabolism and OCCC cells has not been reported.

## 7 Discussion

As described previously, LDs play multiple roles in malignancy. In this article, we have presented two OCCC characteristics regarding LDs, namely, cell growth and lipophagy, through experiments using the OCCC cell lines OVSAYO and OVISE. The former is consistent with a reported ccRCC cell phenotype while the latter is not.

ccRCC is a major histological subtype of kidney cancer. Most ccRCC cells lack *VHL* gene function, leading to constitutive expression of hypoxia-inducible factors (HIFs). Consequently, these cells exhibit hypoxia-driven phenotypes associated with FAO suppression and LD generation (Figure 2H). The importance of the hypoxia response is also true for OCCC cells with intact *VHL* function (Ackroyd et al., 2023), because genomic alterations are shared between OCCC and ccRCC and the HIF pathway is more active in OCCC than in other histological subtypes of EOC (Ji et al., 2018). Our data showing that FAO inhibition can augment OCCC cell growth under normoxia with increased LD levels is consistent with recent studies (Du et al., 2017; Xu et al., 2020; Zhou et al., 2023) showing that lipid storage, rather than lipid consumption, is predominant in ccRCC progression (Figure 2H). It will be interesting to determine whether this metabolic trend can be changed reversibly (Figure 2H) depending on tumor conditions such as hypoxia and poor nutrient supply. Further studies are also needed to clarify the molecular mechanisms that define the relative importance of LD generation and FAO across different cancer types.

Our data further indicated that the autophagy activator MAP1S does not contribute to lipophagy-driven LD catabolism in OCCC cells (Figure 2I). These findings are inconsistent with the importance of MAP1S for autophagy-driven LD clearance in ccRCC cells (Xu et al., 2016). Moreover, unlike our previous data (Koizume et al., 2022), neutral lipolysis via hormone-sensitive lipase is responsible for LD catabolism in ccRCC cells under SSH (Ackerman et al., 2018). It remains unclear how cells utilize these different lipolysis mechanisms and whether SSH-driven LD clearance in OCCC cells can reversibly involve neutral lipolysis depending on the cell culture conditions (Figure 2I). Answers to these issues await future investigations.

Compared with ccRCC, biological information on OCCC is currently scarce, possibly because unlike the serous carcinoma subtype, OCCC is a relatively rare cancer type, especially in Western countries. It is thus currently unclear if OCCC is a lipid-dependent cancer type, similar to ccRCC. OCCC is resistant to standard platinum- and taxane-based chemotherapies, but inhibition of EZH2 histone methyl-transferase has been proposed as an effective synthetic lethal therapy in *ARID1A*-mutated OCCC cases (Bitler et al., 2015). However, information on the relationship between PARPi therapy and lipid metabolism is currently limited for ccRCC and totally unclear for OCCC. Thus, exploration of how LDs contribute to common drug resistance mechanisms between these cancer types may represent another future research direction. Furthermore, ccRCC cells in tumors are under hypoxic mimetic conditions, even under normoxia as described above. Information on the hypoxia status in kidney tissues and ccRCC tumors is currently poor, and limited studies have shown that hypoxic regions exist within the normal renal medulla (Brezis and Rosen, 1995) and renal tumors (Little et al., 2018). The adaptive response mechanisms of ccRCC cells to real-hypoxia conditions and associated drug-resistance thus also remain unclear, but their similarity to OCCC cells warrants further investigation.

The OVSAYO cell line used in our present study and previous studies may have a limitation in its histological origin because genomic analysis revealed that this cell line may be derived from serous carcinoma (Anglesio et al., 2013). However, approved therapeutic strategies for ccRCC have been translated into OCCC treatment (Ji et al., 2018). Investigations into LDs in OCCC cells has just started. We expect that active exploration of OCCC characteristics, including cellular lipid storage, will lead to not only a wealth of information regarding the similarities between OCCC and ccRCC beyond morphology but also the development of common promising treatment options targeting identical metabolic routes.

## Data Availability

The raw data supporting the conclusion of this article will be made available by the authors, without undue reservation.
